# Emergency department utilization of case-managed patients following benign gynecologic surgery

**DOI:** 10.1186/s12245-020-00291-z

**Published:** 2020-06-26

**Authors:** Elizabeth A. Kelly, Cara C. Keller, Megan R. Sax, Rocco A. Rossi

**Affiliations:** Department of Obstetrics and Gynecology, University of Cincinnati Medical Center, Medical Sciences Building Room 7264, 231 Albert Sabin Way, Mail Location 0526, Cincinnati, Ohio USA

**Keywords:** Case management, Emergency department visit, Gynecologic surgery, Postoperative complications

## Abstract

**Background:**

Case management has been shown to reduce the amount of unnecessary emergency department visits among Medicaid or uninsured patients. This study aims to determine whether case management is associated with decreased unnecessary emergency department visits among benign gynecology surgical patients in the first 30 days following surgery.

**Results:**

Out of 875 patients, there were a total of 58 return visits to the emergency department within 30 days and only 6 readmissions. Twenty-four emergency department visits occurred in the case-managed group, and thirty-eight emergency department visits occurred in the non-case-managed group. The two factors that were statistically significant for increase odds of return to the emergency department were the type of surgery (inpatient versus outpatient) and case management. The odds for returning to the emergency department for those not receiving case management was found to be 4.53 to that of the case-managed group when controlling for BMI, age, marital status, and type of surgery.

**Conclusion:**

In an effort to reduce healthcare costs, case management is a promising intervention to help postoperative patients manage their care while minimizing emergency department visits.

## Background

Reducing non-urgent emergency department use is an important aspect of reducing the overall cost of healthcare. Previous studies have demonstrated that Medicaid beneficiaries have an almost two-fold higher rate of emergency department (ED) utilization than patients who are commercially insured [1, 2]. Non-urgent (ED) visits result in unnecessary and extraordinary costs to patients, insurers, and institutions [3]. Increasing opportunities for patients to speak with medical care providers after typical business hours may provide patients with an alternative option to presenting to the ED for non-urgent concerns. In fact, a 2010 study projected 4.4 billion dollars in annual savings if non-urgent ED visits were cared for in retail clinics or urgent care centers during the hours these facilities are open [3].

Manners to decrease utilization of the ED by a Medicaid insured population have been suggested and studied, including the use of case managers to assist patients in triaging their medical needs prior to arriving at a healthcare facility. One such study within a primary care venue in Georgia demonstrated a cost savings of 7.3 million dollars over 3 years via the use of case managers [1, 4]. While case management shows the potential for great benefit for diminishing ED utilization in primary care, it has been less studied for postoperative patients [5]. This study aims to evaluate the utility of perioperative case managers assigned to benign gynecologic patients to reduce ED visits postoperatively.

## Methods

The study was conducted at the University of Cincinnati Medical Center and was approved by the University of Cincinnati Institutional Review Board: Project #2014-2780, approved on February 13, 2015. Billing data was used to identify women who received care at either the Center for Women’s Health or the Division of Community Women’s Health and underwent surgery for benign gynecologic indications between January 2013 and December 2014 were included. The Center for Women’s Health is a resident—and advanced practice nurse (APN)—based clinic located in the building adjacent to the University of Cincinnati Medical Center (UCMC). The Division of Community Women’s Health has ten associated health centers spread throughout the community served by APN, midwife, and physician providers. Women who were served by a surgeon based in the Division of Community Women’s Health are given a case manager in the postoperative period, whereas women based out of the Center for Women’s Health do not have case managers for gynecologic surgeries.

This study is an observational cohort study using a retrospective chart review. Demographic data was collected in regard to all patients that met inclusion criteria. Women were included in the study if they had surgery at UCMC in 2013 or 2014 and were either on Medicaid or uninsured. Variables known to be related to an increase in complications or with a plausible explanation for ED return were used in the analysis (Table 1).

**Table 1:** Characteristics of study population

Characteristic	Case-managed		***P*** value
	Yes *n*(675)=(%)	No *n*(200)=(%)	
**BMI**			0.2124
< 30	285 (43.64)	108 (55.67)	
≥ 30	368 (56.63)	86 (44.33)	
**Marital status**			0.2524
Married	115 (17.14)	30 (15.0)	
Not Married	556 (82.86)	170 (85.0)	
**Average age**	40.24 (StdDev ± 11.25)	36.4 (StdDev ± 13.1)	0.0059
**Race**			0.9305
White (non-Hispanic)	176 (26.11)	54 (27)	
Black (non-Hispanic)	429 (63.65)	135 (67.5)	
Hispanic	50 (7.42)	3 (1.5)	
Other	19 (2.82)	8 (4)	
**Residence**			0.7730
Adjacent to hospital zip code	137 (20.63)	40 (20)	
Other	527 (79.37)	160 (80)	
**Insurance status**			0.1421
State insurance/self-pay	614 (91.37)	199 (99.5)	
Private insurance	58 (8.63)	1 (0.50)	
**Surgery type**			0.0049
Same-day surgery	473 (70.28)	164 (82.41)	
Inpatient	200 (29.72)	35 (17.59	

Table 1 includes demographics of patients included in the study, whether they were case-managed, and who returned to the emergency department

The primary outcome for this study was to return to the ED within 30 days of gynecologic surgery. The patient characteristics of the case-managed group were compared to a referent group of non-case-managed patients. Significant differences were defined as comparisons with a probability value of *P* < 0.05 and a 95% confidence interval not inclusive of the null value of 1.0. After univariate logistical regression was performed, stepwise backward elimination determined the final model.

Statistical analysis was performed using SAS software version 9.4. Variables were assessed for the presence of confounding by the risk factors that are known to be associated with return to the ED. The final unique model for each variable was then chosen with a resulted adjusted odds ratio, *P* value, and 95% confidence interval. This study was powered to detect an odds ratio of 1.5 or greater. For the final model, we used logistic regression with stepwise backward elimination.

## Results

Of 875 total patients identified, 675 who were case-managed and 200 were not case-managed. The BMI, marital status, and race of the two groups were not significantly different. The zip code of the patient’s listed address was also collected given that patients are more likely to go to an ED closest to their residence and since the community health centers are spread throughout the county, the patients attending these health centers may be more geographically diverse. The zip codes were then simplified into those adjacent or within the zip code of the hospital and those outside of the adjacent or hospital zip codes. The patient’s residential zip code did not demonstrate statistical significance. The mean age between the two groups was 40 for the case-managed group and 36 for the non-case-managed group. This was statistically significant with a *P* value < 0.05. The other statistically significant factor was the surgical type with same-day surgery being more common in the non-case-managed group.

In Table 2, the surgical complications are listed. These were not statistically significant differences among the two groups with an overall complication rate of only 3.5% in total when accounting for intra-operative organ damage, reoperations, venous thromboembolism, death, unplanned admission to intensive care unit, infection, and transfusion of two or more units.

**Table 2:** Surgical characteristics of study population

Surgical characteristic	Case-managed		***P*** value
	Yes *n*(675)=(%)	No *n*(200)=(%)	
Unplanned intensive care admission	2 (0.30)	0 (0)	0.9844
Intraoperative organ damage	5 (0.74)	1 (0.5)	0.0147
Death	0	0	
Venous thromboembolism	0	0	

Table 2 demonstrates the surgical characteristics between patients who were and were not case-managed

In total, 59 patients returned to the ED, for a return rate of 6.7% (59 out of 875 women). Six women returned twice and one woman returned three times.

In the case-managed group, there were 29 single return visits to the ED and no one came back more than once. In the non-case-managed group, there were 23 single return visits to the ED, 6 people who returned twice and one who returned three times. The ED utilization rate among the non-case-managed group was 15% for individual utilization of the ED. The case-managed group ED utilization was 3.7%.

When controlling for variables for ED admission, only case management and surgical type demonstrated statistical significance. The odds of returning to the ED after inpatient surgery is 3 times that of after having a same-day surgery. Case management rendered an adjusted odds ratio of 0.235, the odds of returning for those that were not case-managed. When controlling for other variables, the odds for returning to the ED for those not receiving case management was found to be 4.531 that of the case-managed group when controlling for BMI, age, marital status, and type of surgery (Table 3).

**Table 3:** Logistic regression of characteristics associated with emergency department visits

	Unadjusted			Adjusted		
Characteristic	Odds ratio	CI	***P*** value	Odds ratio	CI	***P*** value
Case management	0.240	0.135–0.428	< 0.0001	0.235	0.132–0.416	< 0.0001
BMI ≥ 30	0.713	0.419–1.213	0.2124	0.823	0.459–1.476	0.5137
Unmarried	1.641	0.840–3.203	0.1469	1.627	0.835–3.171	0.1526
Age	0.982	0.957–1.008	0.1722	0.982	0.957–1.007	0.1531
Inpatient surgery	3.804	1.712–5.558	0.0002	3.062	1.707–5.495	0.0002
Residence adjacent to hospital zip code	1.105	0.561–2.178	0.7730	1.108	0.538–2.281	0.7802
Race						
White (non-Hispanic)	Referent					
Black (non-Hispanic)	0.851	0.475–1.524	0.5872	0.891	0.465–1.706	0.7279
Hispanic	0.707	0.200–2.493	0.5893	1.073	0.285–4.039	0.9168

Table 3 shows the adjusted and unadjusted logistic regression statistics of patients who were presented to the emergency department

## Discussion

In this single-center retrospective observational cohort study involving 875 women following benign gynecologic surgery, we found that the odds of returning to the ED within 30 days of surgery are 4.53 times higher if a patient is not case-managed compared to those that receive case management, even when adjusting for other factors. Inpatient surgery also demonstrated statistical significance for return to the ED, with an odds ratio of 3.062.

Several studies have examined case management among various populations; however, none have been studied in patients undergoing surgery for benign gynecologic indications. ED visits are associated with a significant financial burden to the healthcare system, whereas case managers provide a valuable resource at a reduced cost and have been shown to increase patient satisfaction. In this high-risk population, the odds of returning to the ED in the non-case-managed group were found to be 4.531 to that of the case-managed group when controlling for BMI, age, marital status, and type of surgery.

The limitations of our study are the inability to access records from outside emergency departments; however, given that the geographic distribution of the patients is similar, one may infer that the rate of return to an ED other than UCMC would be similar between the groups. Given that case managers are in regular contact with their patients when an issue arises, the bias in this case would seem to err on leading more of the case-managed patients to return to UCMC ED if presenting with a complaint. Another limitation is the absence of data on other co-morbidities that may confer a higher risk for patients to present to the ED, such as diabetes or smoking status. This will be an area in which to expand in the future.

The strengths of our study include the completeness of the data with less than 2% missing. Another strength is that all the demographic, visit information, and surgical complications were collected through the electronic health records by one of the authors. Our study also included a large number of participants at a single institution where operative practices are similar and ED protocols and triage are established. In conclusion, case management of postoperative patients demonstrates a promising correlation in decreasing ED visits within 30 days of surgery for benign gynecologic indications.

## Conclusion

Reducing the annual number of non-urgent ED visits may improve unnecessary medical spending for patients, insurers, and institutions [3]. While prior studies have demonstrated reduced non-urgent ED visits among patients in the primary care setting who have accessible case managers, this study demonstrates a similar pattern in postoperative patients [5]. Initiating case management programs for postoperative patients significantly improves the utilization of care and medical costs for patients and providers.

Although this study demonstrates that case management may decrease unnecessary utilization of ED for postoperative patients, further research should address the ideal length duration of follow-up for case management following surgery, and whether the role of case managers is equally significant in privately insured patients. Ultimately, more data, such as in the form of a randomized controlled trial, will aid in validating the use of case managers to decrease the risk of unnecessary ED presentation.

## Data Availability

The data that support the findings of this study are available from Dr. Rocco Rossi, but restrictions apply to the availability of the data, which were used under license for the current study, and so are not publicly available. Data are however available from the authors upon reasonable request and with permission of Dr. Rocco Rossi.

## Abbreviations

ED: Emergency department
APN: Advanced practice nurse
UCMC: University of Cincinnati Medical Center
